# Reliability and validity of the Mentalization Questionnaire in a potentially depressed group of Chinese college students

**DOI:** 10.3389/fpsyt.2025.1555549

**Published:** 2025-07-07

**Authors:** Xin Huang, Zemin Zhou, Guang Yang, Jiao Liu, Xuan Li, Xuemei Li

**Affiliations:** ^1^ Psychology Teaching and Research Office, Hunan University of Medicine, Hunan, China; ^2^ Clinical Psychological Counseling and Intervention Center, The Third People’s Hospital of Guang’an, Sichuan, China; ^3^ Department of psychosomatic, The First Afiliated Hospital of Kangda College of Nanjing Medical University/The First People’s Hospital of Lianyungang, Jiangsu, China; ^4^ Department of Psychiatry, Dianjiang People’s Hospital of Chongqing, Chongqing, China; ^5^ Department of Psychiatry, Key Laboratory of Major Brain Disease and Aging Research (Ministry of Education), The First Affiliated Hospital of Chongqing Medical University, Chongqing, China

**Keywords:** mentalization, MZQ, depression, reliability, validity

## Abstract

**Purpose:**

Mentalization, as a core psychosocial function, not only encompasses emotional regulation but also involves the perception and comprehension of one’s own and others’ emotional states, constituting a crucial capacity for establishing adaptive interpersonal relationships. University students, due to their unique challenges including academic competition, identity transition during this critical developmental period, and social pressures, demonstrate that impairments in mentalization capacity may significantly elevate the risk of depressive disorders. Concurrently, the exacerbation of depressive symptoms can further compromise mentalization functioning, thereby creating a self-perpetuating pathological cycle.However, there is a lack of validated assessment tools for mentalization in China. This study aims to provide a validated instrument for assessing mentalization in the Chinese population.

**Methods:**

The Chinese version of the Mentalization Questionnaire (MZQ) was developed through rigorous cross-cultural adaptation procedures. Following Brislin’s translation model, medical English experts initially translated the instrument into Chinese. Subsequently, a panel of specialists in psychology and psychiatry conducted comprehensive reviews, back-translation, and iterative revisions to ensure conceptual equivalence. The finalized scale was administered to 874 Chinese university students exhibiting subthreshold depressive symptoms (Patient Health Questionnaire-9 [PHQ-9] score ≥10). Exploratory factor analysis (EFA) and confirmatory factor analysis (CFA) were sequentially implemented to establish and verify the factorial structure. Concurrent validity was examined using the 8-item Reflective Functioning Questionnaire (RFQ-8). To assess test-retest reliability, 85 participants were randomly selected for retesting one week after initial administration.

**Results:**

The MZQ revealed a 3-factor structural model, and confirmatory factor analysis showed satisfactory fit indices for all three structures (χ²/df = 3.69, NFI = 0.83, CFI = 0.87, GFI = 0.92, IFI = 0.87, TLI = 0.83, RMSEA = 0.078). The correlation coefficient between the total MZQ score and the RFQ-C (Certainty about mental states) was -0.557, while the correlation coefficient between the total MZQ score and the RFQ-U (Uncertainty about mental states) was 0.428.

**Conclusion:**

The Chinese version of the Mentalization Questionnaire (MZQ) demonstrates good validity and reliability, making it a suitable tool for assessing mentalization levels among college students with potential depressive symptoms.

## Introduction

Depression is one of the most prevalent mental disorders among university students globally. In China, the detection rate of depressive symptoms among college students has reached 24.7% (1), with recent studies indicating a rising trend (2). Depression not only leads to academic performance decline (3)and impaired social functioning (4), but is also significantly associated with an increased risk of suicide (5).

The concept of mentalization originates from psychoanalysis and psychodynamic psychotherapy, developed by Fonagy, Bateman, and colleagues (6). Mentalization is a psychological process that refers to the ability to understand one’s own and others’ mental states by recognizing and reflecting on emotions, behaviors, and intentions (7). Research has shown that deficits in mentalization—including lack of emotional awareness, insufficient self-reflection, or conflating internal mental states with external reality (8)—may be closely linked to adverse childhood experiences or disordered attachment patterns (9). The development of mentalization abilities has been found to positively mitigate the effects of childhood trauma and support individuals in coping with early adversity (10), thereby reducing the risk of mental disorders (11). Increasing evidence suggests that mentalization deficits may contribute to the development of depressive symptoms (12). In clinical samples of adolescents, deficiencies in mentalization abilities have been associated with the severity of depression and partially explain the link between childhood trauma and depressive symptoms (13). Severe chronic and/or treatment-resistant depression has also been linked to more pronounced mentalization deficits (14). In terms of psychotherapeutic processes and outcomes, mentalization is a central focus of psychodynamic approaches and has been shown to be a crucial mediating and moderating factor (15, 16). Additionally, mentalization is recognized as a protective psychological resource; improvements in mentalization enable individuals to develop a more integrated and coherent sense of self, enhancing their ability to manage interpersonal and psychological challenges, and fostering the development of healthy, harmonious relationships (17).

Fonagy and colleagues developed therapies aimed at enhancing mentalization abilities, known as Mentalization-Based Therapy (MBT), which was initially designed for the treatment of borderline personality disorder. MBT is now also applied to antisocial personality disorder, substance abuse, eating disorders, as well as family therapy, adolescent psychotherapy, and school and social group therapy, all of which have demonstrated significant efficacy (6). In the process of MBT, it is necessary to measure changes in mentalization levels to evaluate the effectiveness of the treatment. Most mentalization assessment tools are clinician-rated scales or semi-structured interviews, such as the Adult Attachment Interview (AAI) and the Reflective Function Scale (RFS). These tools require specialized training for the evaluator, making them relatively complex and time-consuming to administer.

In recent years, mentalization therapy has gradually been introduced in China. Research indicates that mentalization therapy can alleviate negative emotions such as anxiety and depression in individuals addicted to methamphetamine and reduce their chronic cravings for drugs (18). For adolescent patients with depression and their mothers, family-based mentalization therapy has been shown to reduce anxiety and depression symptoms, enhance mentalization abilities, and improve the parent-child relationship (19). However, research on mentalization measurement in China is still in its early stages, with a limited number of available assessment tools. The Reflective Functioning Questionnaire (RFQ-8), developed by Fonagy’s team, is the only self-report scale for assessing adult mentalization levels (20) and was introduced to China by Xu Lisi’s team.

Although the Chinese version of RFQ-8 has been applied among Chinese university student populations (21), its validity remains significantly controversial. Research indicates that the excessive mentalization subscale (RFQ-C) of RFQ-8 lacks sufficient validity (22) and can only measure two dimensions of mentalization (excessive mentalization and deficits), failing to cover critical dimensions such as psychic equivalence mode and emotion regulation, which may lead to the neglect of key therapeutic targets (23). Furthermore, the item design of RFQ-8 is based on Western cultural contexts and may fail to capture the influence of emotional restraint and collectivist tendencies in Chinese culture on mentalization, potentially causing patients to conceal genuine emotions and resulting in an underestimation of mentalization deficits when using Western scales.

To provide a simple and easy method for measuring mentalization levels, Hausberg’s team developed the Mentalization Questionnaire (MZQ) among psychiatric inpatients in 2012 (23). The MZQ demonstrates good reliability and validity and is capable of assessing four dimensions of mentalization in individuals with psychological disorders. These four dimensions are:

1. Refusal to self-reflect, which includes avoiding thoughts about internal states or systematically rejecting one’s own feelings due to fear of being overwhelmed.
2. Emotional awareness, which refers to the ability to perceive and differentiate one’s internal emotional states.
3. Psychological equivalence, a pre-mentalization mode of thinking that equates internal mental states with external reality, believing that inner feelings and thoughts are synonymous with reality.
4. Emotional regulation, which encompasses the inability to manage emotions, leading to feelings of helplessness and a sense of being threatened by one’s own emotions.

Compared to the RFQ-8, the MZQ measures more dimensions of mentalization and captures the cognitive, emotional, and affective aspects involved in the mentalization process. Currently, there is no scale available in China to assess the mentalization levels of individuals with depression. Therefore, this study aims to translate, adapt, and evaluate the initial Chinese version of the MZQ. A psychometric evaluation was conducted to test the factorial validity and reliability of the scale using a representative sample. This study seeks to fill the gap in mentalization-related measurement tools in China, providing an effective instrument for assessing the mentalization levels of individuals with depression. Additionally, it aims to enhance the understanding of mentalization levels among Chinese individuals and to offer more objective information and accurate references for psychotherapy.

## Materials and methods

### Measures

#### Chinese Version of the Mentalization Questionnaire

The initial Chinese version of the MZQ, translated by the researchers, consists of 15 items rated on a 5-point Likert scale, with responses ranging from 1 (“not consistent at all”) to 5 (“completely consistent”). It includes four subscales: Refusal to Self-Reflect (4 items: questions 5, 9, 13, and 14), Emotional Awareness (4 items: questions 8, 10, 11, and 15), Psychic Equivalence Mode (4 items: questions 1, 4, 7, and 12), and Regulation of Affect (3 items: questions 2, 3, and 6). The total score ranges from 15 to 75 points, with higher scores indicating lower mentalization ability. The Cronbach’s α coefficient of the scale is 0.81.

#### Reflective Functioning Questionnaire-8 Chinese version

The RFQ-8 consists of 8 items, with response options ranging from “strongly disagree” to “strongly agree.” The ratings are coded as “0, 0, 0, 0, 1, 2, 3” and “3, 2, 1, 0, 0, 0, 0.” The scale includes two subscales: Certainty about Mental States (RFQ-C) and Uncertainty about Mental States (RFQ-U). Hypermentalizing refers to behaviors that involve excessive and overly detailed imagination and reasoning beyond the objective facts. In contrast, hypomentalizing refers to a lack of specific thinking content and form, resulting in an inability to comprehend subtle or complex psychological states of oneself and others. RFQ-C and RFQ-U respectively measure different types of impairments in mentalization, with higher scores indicating greater impairment in reflective functioning.

#### Patient Health Questionnaire-9

The depression levels of the participants were measured using the Patient Health Questionnaire-9 (PHQ-9) (24). The PHQ-9 consists of 9 items, each scored from 0 to 3, resulting in a total score range from 0 to 27. A score of 0 indicates “Not at all,” while a score of 3 indicates “Nearly every day.” Higher scores indicate more severe levels of depression. The scoring ranges are as follows: mild depression (6 to 9 points), moderate depression (10 to 14 points), severe depression (15 to 21 points), and extremely severe depression (22 to 27 points).

### Methods

#### Research design

##### Translation and pre-experimentation of the Mentalization Questionnaire

After obtaining authorization from the original author, the English version of the MZQ was translated into Chinese by medical English experts and introduced in China. The translation process followed Brislin’s scale translation principles. To develop the Chinese version of the MZQ, the translation was reviewed, back-translated, and revised by research experts from psychology, psychiatry, and other related fields to minimize ambiguities caused by cross-cultural differences, written expressions, or other factors (25). A pre-experiment was conducted with 30 ordinary college students, and feedback from the participants was incorporated (26). After several rounds of verification and modification, the final version of the Chinese MZQ was established.

##### Sample selection and grouping

The study subjects were medical students enrolled at Chongqing Medical University, with a total of 17,217 questionnaires distributed. After excluding invalid questionnaires with over 90% repetitive responses, 12,375 valid questionnaires were collected. Ultimately, 874 individuals with PHQ-9 scores ≥10, indicating potential depressive symptoms, were included as the analytical sample. The average age of this sample was 19.76 ± 1.80 years; among them, 642 were male and 232 were female.

This subgroup was selected because extensive research has demonstrated that deficits in mentalization are a core maintaining factor of depressive symptoms (27). University students, being in a critical period of psychosocial development, face multiple stressors such as academic competition and identity formation, resulting in a significantly higher prevalence of depressive symptoms compared to the general population (28). Additionally, these symptoms are often accompanied by impairments in emotion regulation and self-reflection abilities (29).

These 874 individuals were randomly divided into two groups: the exploratory group and the validation group. The exploratory group, consisting of 435 participants, was used to analyze the structure and exploratory factors of the Initial Chinese Version of the MZQ scale. The results of this analysis were used to refine and eliminate items, resulting in the Revised Edition of the Chinese MZQ scale. The validation group, consisting of 439 participants, was used to test the reliability and validity and to conduct confirmatory factor analysis of the Revised Edition of the Chinese MZQ scale.One week after the initial test, a class was randomly selected for a retest, and 94 questionnaires were distributed. After excluding invalid questionnaires with a repetition rate of over 90% for response options, 85 valid questionnaires remained.

The sample size of this study was determined based on the following principles:

Exploratory Factor Analysis (EFA): According to the recommendation by Goldberg and Velicer (30), EFA requires a sample size of at least 5–10 times the number of variables. The Chinese version of MZQ contains 15 initial items, thus the minimum sample size required for EFA is 15×10 = 150 cases. The exploratory group sample size in this study was 435 cases, meeting the minimum requirement.

Confirmatory Factor Analysis (CFA): With reference to the standard proposed by McNeish and Wolf (31), CFA sample size should satisfy at least 10 observations per free parameter. The three-factor model contains 14 items and 3 latent variables, with free parameters totaling 14 + 3 = 17, therefore the minimum sample size is 17×10 = 170 cases. The validation group sample size in this study was 439 cases, fulfilling this requirement.

Retest Reliability: Based on Weir’s (32) sample size calculation formula (α=0.05, β=0.20, expected reliability coefficient=0.7), the estimated minimum required sample size was 52 cases. The test-retest sample size in this study was 85 cases, meeting the requirement.

##### Validity and reliability testing methods

#### Validity test

##### Structural validity

Exploratory factor analysis (EFA):KMO test and Bartlett spherical test were conducted on the sample of the exploration group, and the maximum variance rotation method was used to extract factors, and items were selected according to the factor loadings and theoretical consistency.

Confirmatory factor analysis (CFA): The fit of the three-factor model was tested on the validation group sample, and the rationality of the model was evaluated by standardized regression coefficients and fitting index.

Conventional validity: The total score of MZQ was calculated with the Chinese version of Reflective Function Questionnaire (RFQ-8) as the conventional validity tool, and the correlation coefficient between RFQ-C (certainty scale) and RFQ-U (uncertainty scale) was calculated.

#### Reliability test

Internal consistency: Calculate the total table and Cronbach’s α coefficient of each dimension to evaluate the consistency between items.

Retest reliability: one week after the initial test, a class was randomly selected for retest to test the stability of the scale through the correlation coefficient within the group.

### Statistical methods

SPSS 25.0 was used for item analysis, reliability analysis, exploratory factor analysis, and correlation analysis. Confirmatory factor analysis was conducted using AMOS 25.0.

## Results

### Item analysis

The results of the critical ratio method showed that all items had significant differences between the high and low groups (P < 0.001), with critical values ranging from 7.016 to 15.064. Correlation analysis results indicated that the correlation coefficients between each of the 15 items and the total scale score ranged from 0.493 to 0.672, all of which were statistically significant (P < 0.001). After deleting item 13, the Cronbach’s α coefficient increased to 0.829, which is higher than the original coefficient; therefore, item 13 was removed. The Cronbach’s α coefficients of the remaining items ranged from 0.812 to 0.822, and further deletion did not increase the coefficient, indicating that the items in the scale are highly differentiated and reasonably designed.

### Validity analysis

#### Construct validity

After removing item 13, an Exploratory Factor Analysis (EFA) was conducted on the Chinese version of the MZQ scale using the exploration group to examine its structural validity. The results revealed a KMO value of 0.847, and the Bartlett’s test of sphericity yielded χ² = 1514.918 (P < 0.001), indicating that the data were suitable for factor analysis. Using the maximum variance rotation method, all factor loadings were above 0.4, and four principal components were extracted. The total explained variance reached 63.03%, leading to a four-factor structural model. However, since item 2 had double loadings on both factor 1 and factor 4, it was more appropriate to assign item 2 to factor 1. If item 14 were classified into factor 4 independently, the factor would be difficult to define and name. Therefore, item 14 was classified into factor 2 based on theoretical considerations. See **Table 1** for details.

**Table 1:** Exploratory factor analysis results of the Chinese version of Mentalization Scale (MZQ) clinical sample group (n=435).

Item	Factor loading(Rotated)			
	Factor1	Factor2	Factor3	Factor4
1	0.773	0.161	0.017	-0.019
2	0.456	-0.059	0.094	0.562
3	0.62	0.158	0.316	0.148
4	0.625	0.138	0.107	0.262
5	0.269	-0.011	0.551	0.418
6	0.175	0.294	0.609	0.022
7	-0.141	0.166	0.759	0.111
8	0.352	0.165	0.62	0.077
9	0.412	0.346	0.471	-0.142
10	0.323	0.664	0.172	0.048
11	0.023	0.76	0.245	0.05
12	0.351	0.577	0.133	0.093
14	0.044	0.271	0.075	0.795
15	-0.024	0.615	0.128	0.438

Validity factor analysis was conducted to verify the three-factor structure explored. The results showed χ² = 273.38, χ²/df = 3.69, P < 0.001, with NFI = 0.83, CFI = 0.87, GFI = 0.92, IFI = 0.87, TLI = 0.83, and RMSEA = 0.078. The chi-square degree of freedom ratio is less than 5, indicating that the model is compatible with the data; RMSEA is close to the critical value of 0.08, indicating that the model fitting is acceptable; CFI and NFI are slightly lower than the ideal value of 0.90, but still within a reasonable range in exploratory research.

**Figure 1:**
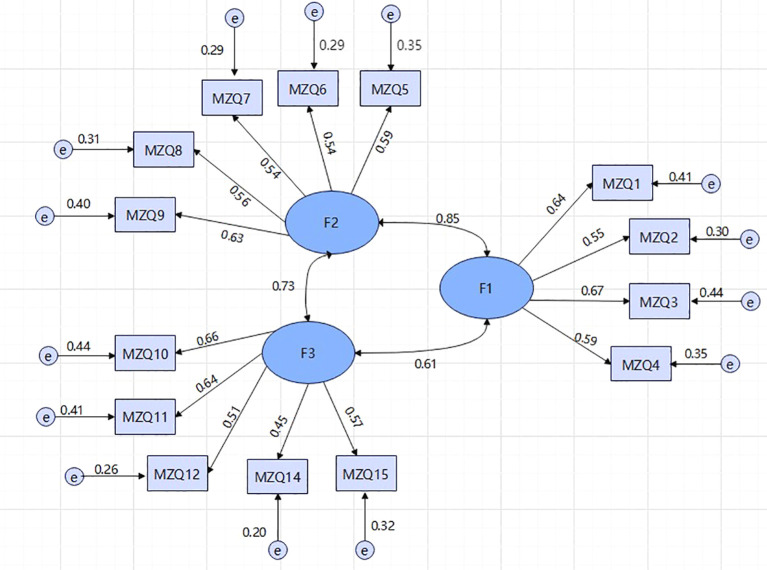
Three-Factor Model of the MZQ in a Potentially Depressed Group of Chinese College Students.

The standardized regression weights of the 14 items ranged from 0.446 to 0.667, all above the threshold of 0.40 (see **Figure 1**). The composite reliability (CR) values of each factor were all above 0.7, indicating that the measurement indicators within the factors were well extracted and demonstrated good internal consistency. The square roots of the AVE values for the three factors ranged from 0.564 to 0.614, and the Pearson correlation coefficients between factor 1, factor 2, and factor 3 were between 0.445 and 0.595, all smaller than the AVE values for each factor, indicating satisfactory discriminant validity among the three factors.In general, although the three-factor model is not perfect, it can effectively reflect the core dimensions of Chinese college students’ psychological ability.

After review by two researchers, the factors were renamed, resulting in a final three-factor model: Factor 1 was named “Cognition and Communication,” including items 1 to 4; Factor 2 was named “Self-Reflection and Emotion Regulation,” including items 5 to 9; and Factor 3 was named “Self-Emotional Perception and Sensitivity,” including items 10, 11, 12, 14, and 15. See **Table 2**.

**Table 2:** Confirmatory factor analysis results of the Chinese version of Mentalization Scale (MZQ) in the validation group (n=439).

Factors	Item	Standardized Regression Weights	AVE	Square root of AVE value	CR
Factor1	MZQ1	0.638	0.377	0.614	0.705
	MZQ2	0.547			
	MZQ3	0.667			
	MZQ4	0.593			
Factor2	MZQ5	0.591	0.327	0.572	0.708
	MZQ6	0.538			
	MZQ7	0.542			
	MZQ8	0.556			
	MZQ9	0.629			
Factor3	MZQ10	0.662	0.318	0.564	0.704
	MZQ11	0.643			
	MZQ12	0.512			
	MZQ15	0.568			
	MZQ14	0.446			

#### Criterion validity

The total score of the Chinese version of the MZQ scale and the dimension scores represented by the three factors in the clinical sample group were negatively correlated with the RFQ-C score, with correlation coefficients ranging from -0.376 to -0.557. Conversely, a positive correlation was observed with the RFQ-U, with correlation coefficients between 0.306 and 0.428. These results indicate that the Chinese version of the MZQ scale can, to a certain extent, reflect the degree of mentalizing deficits. See **Table 3** for details.

**Table 3:** Correlation between the measurement results of the Chinese version of the Mentalization Scale (MZQ) in depression group samples and scores of other scales number.

Variable	MZQ Total Score	Factor 1 Cognition And Communication	Factor 2 Self-Reflection	Factor3 Self-Emotional Perception and Sensitivity	RFQ-C	RFQ-U
MZQ Total Score	1***					
Factor 1 Cognition And Communication	0.774***	1***				
Factor 2 Self-Reflection	0.869***	0.582***	1***			
Factor3 Self-Emotional Perception and Sensitivity	0.802***	0.343***	0.568***	1***		
RFQ-C	-0.557***	-0.376***	-0.445***	-0.529***	1***	
RFQ-U	0.428***	0.306***	0.337***	0.395***	-0.291***	1***

***P<0.01.

### Reliability test

#### Internal consistency reliability

The Cronbach’s α coefficient of the Chinese version of the MZQ scale is 0.839, and the Cronbach’s α coefficients for the three dimensions—factor 1, factor 2, and factor 3—are 0.702, 0.708, and 0.694, respectively, indicating good internal consistency.

#### Retest reliability

The results of the retest indicate that the reliability coefficient for the total score of the three-factor structure model is 0.698. The test-retest reliability coefficients for the three subscales—factor 1, factor 2, and factor 3—are 0.524, 0.654, and 0.611, respectively, demonstrating good retest reliability.

## Discussion

In this study, the Chinese version of psychologized questionnaire (MZQ) was developed through cross-cultural adaptation and psychometric validation, and its good reliability and validity were confirmed in a group of Chinese college students with sub-threshold depressive symptoms.

The three-factor structure of the Chinese version of MZQ (cognitive and communication, self-reflection and emotion regulation, self-emotion awareness and sensitivity) differs significantly from the original four-factor model (23). This difference may reflect unique expressions of psychologization in the context of Chinese culture. For example, “refusing self-reflection” and “psychological equivalence” in the original scale were merged into “cognitive and communication” in this study, which may be closely related to the tendency for emotional suppression in collectivist cultures. Chinese society emphasizes interpersonal harmony and “face preservation” (33), leading individuals to express internal conflicts through indirect communication rather than directly rejecting reflection or blurring the boundaries between psychology and reality (34). This finding is consistent with recent cross-cultural studies: Raimondi et al. (35) found that the Italian version of MZQ retained a four-factor structure, while tools in Asian cultures (such as the elderly depression screening tool developed by 36) tend to integrate the dimensions of emotion regulation and interpersonal interaction, suggesting that cultural values may reshape the operational definition of psychologization. In addition, the “self-emotion awareness and sensitivity” factor in this study encompasses over-arousal to threatening interpersonal signals, such as item 12, which may be related to the “highly sensitive coping style” formed by Chinese college students in highly competitive environments (37). In contrast, Western research places more emphasis on the association between psychogenic deficits and early trauma (10), while in this sample, emotion awareness issues may be influenced by both social pressures and cultural norms.

The three-factor model of the Chinese version of MZQ strikes a balance between simplicity and cultural specificity. Compared to RFQ-8, its advantage lies in capturing multidimensional psychogenic deficits: RFQ-8 can only distinguish between “excessive psychologization” and “psychogenic deficits” (38), whereas MZQ reveals the central role of emotional regulation in psychogenicity through the “self-reflection and emotion regulation” factor (with a strong negative correlation with RFQ-C, r= -0.557). This finding supports Christensen et al. (39)’s discovery that emotional regulation disorders may serve as a mediating mechanism for psychogenic deficits in depressive populations. The comparison with the international version of MZQ further highlights the necessity of cultural adaptation adjustments. For example, Hausberg et al. (23) reported a CFI of 0.92 for the original scale, while in this study, the CFI was 0.87, slightly below the ideal value. This difference may stem from item deletion, such as removing item 13, or the impact of culture on factor loadings. A similar phenomenon is observed in Riedl et al. (40)’s MZQ-6 study: although the shortened version improved clinical utility, its simplified factor structure may have sacrificed some culturally sensitive dimensions. This study achieved a better balance between model simplicity and cultural validity by retaining key cultural features, such as items related to emotional suppression.

This study provides empirical support for the cross-cultural applicability of psychologization theory. Traditional psychologization theory emphasizes the universality of four dimensions (6), but this result suggests that in collectivist cultures, the boundaries of psychologization may need to be redefined. For example, the introduction of the “cognition and communication” factor challenges the assumption that “self-reflection is rejected” as an independent dimension, indicating that cultural values may influence psychologization performance by integrating cognitive and emotional processes (41). This finding aligns with Parolin et al. (17)’s argument that the development of psychologization capabilities must consider how cultural norms shape self-other representations. In clinical practice, the Chinese version of MZQ can provide precise targets for depression intervention. For example, individuals with low “self-emotion awareness” scores may benefit from emotion labeling training in MBT (42), while those with “cognitive and communication” deficits need to strengthen their psychological skills in interpersonal interactions. Additionally, the combined use of MZQ and PHQ-9 can achieve a dual screening of “symptoms-mechanisms,” providing an operational assessment framework for mental health services on campus.

This study has the following limitations: First, the sample is limited to medical students, whose professional stress may amplify emotional suppression tendencies (such as the high load in item 5), and future research needs to verify its generalizability in non-medical populations. Second, there is a lack of discriminative validity data for clinical depression patients, and further comparisons are needed to assess the sensitivity differences between MZQ in subclinical symptoms and clinical diagnosis. Methodologically, mixed methods (such as interviews) can be combined to delve deeper into the cultural implications of the three factors (31), for example, how Chinese students interpret the differences in the expressions “refusing self-reflection” and “psychological equivalence”.

## Conclusion

This study confirms that the Chinese version of the MZQ has good reliability and validity among college students with potential depression. Its three-factor structure (cognition and communication, self-reflection and emotion regulation, self-emotion awareness and sensitivity) reflects the core dimensions of psychological competence in a Chinese cultural context. As the first integrated tool for psychological assessment that combines emotion regulation and cross-cultural adaptation, the MZQ provides an efficient means for mental health screening on campus. Future research should validate its discriminant validity in clinical populations and explore its integration with MBT interventions to promote the practical application of the “assessment-intervention” loop.

## Data Availability

The original contributions presented in the study are included in the article/supplementary material. Further inquiries can be directed to the corresponding author.

## Appendices The Chinese verison of MZQ.

**Table:** Please select the option that best reflects your true feelings and actual situation.

Dimension	Item	Strongly Disagree	Somewhat Disagree	Neither Agree nor Disagree	Somewhat Agree	Strongly Agree
**Cognition** **& Communication**	1. If I think I am about to be criticized or offended, my fear intensifies.	1	2	3	4	5
	2. Others’ analyses do not help me understand my own feelings.	1	2	3	4	5
	3. Emotions sometimes feel dangerous to me.	1	2	3	4	5
	4. I only believe someone genuinely likes me if I have sufficient concrete evidence (e.g., dates, gifts, or hugs).	1	2	3	4	5
**Self-Reflection &** **Emotion Regulation**	5. I think it’s best not to have any feelings most of the time.	1	2	3	4	5
	6. I often cannot control my emotions.	1	2	3	4	5
	7. I find it hard to believe that relationships can change.	1	2	3	4	5
	8. I tend to ignore feelings of physical tension or discomfort until they become so intense that they overwhelm me.	1	2	3	4	5
	9. Talking about feelings makes them feel stronger.	1	2	3	4	5
**Self-Emotion Awareness** **& Differentiation**	10. Sometimes I only realize how I felt afterwards, upon reflection.	1	2	3	4	5
	11. I often have difficulty fully sensing/perceiving my own feelings.	1	2	3	4	5
	12. The thought of someone criticizing or offending me often makes me feel threatened.	1	2	3	4	5
	14. Most of the time, I don’t want to talk to others about my thoughts and feelings.	1	2	3	4	5
	15. I am often unaware of what is happening inside me.	1	2	3	4	5
